# Fenestration Labioreduction of the Labium Minus: A New Surgical Intervention Concept

**DOI:** 10.1155/2014/671068

**Published:** 2014-02-04

**Authors:** Adam Ostrzenski

**Affiliations:** Institute of Gynecology, Inc., 7001 Central Avenue, St. Petersburg, FL 33710, USA

## Abstract

*Objectives*. To test applicability of the new surgical concept for labioreduction of the labia minora. *Study Design*. The observational, prospective, case series study was designed. *Subjects*. Three consecutive subjects were included. *Methods*. The application of new surgical intervention was tested. *Main Outcome Measures*. A primary outcome measured applicability of the fenestration labioplasty and secondary measures was used to evaluate surgical resolution of medical, emotional, and social symptoms; aesthetic outcomes; and potential complications. *Results*. Symptomatic, asymmetrical, and enlarged labia minora were associated with aesthetic dissatisfaction from deformations of the labia minora. The new operation was applied easily and without complications. The procedure reduced height and length, accomplished symmetries, preserved natural color and contour, and accomplished the labium minus expected appearance. Postoperatively, physical, emotional, social symptoms and signs resolved, pleasing surgical outcomes exceeded subjects' aesthetic expectations. Meaningfully, self-image and self-confidence improved in all subjects. No feelings of regrets were reported. Emotional tensions were reduced, social openness improved, intimate interaction increased, and their body image perception improved following the operation. *Conclusion*. In this study group, fenestration labioreduction with inferior flap transposition was easy to execute without complications and the method was reproducible; the new operation achieves pleasing aesthetic results and the procedure improves physical, emotional, and social wellbeing.

## 1. Introduction 

In September 2007, the American College of Obstetricians and Gynecologists (ACOG) in its committee's opinion recommended that labium minus operations can be performed to alter the size or shape (labioreduction) for the following medical indications: “labial hypertrophy or asymmetrical labial growth secondary to congenital conditions, chronic irritation, or excessive androgenic hormones.” Additionally, the ACOG Committee Opinion suggested that “clinicians who receive request from patients for such procedures should discuss with the patient the reason for her request and perform an evaluation for any physical signs or symptoms that may indicate the need for surgical intervention.” Also, ACOG viewed that women should be discouraged from cosmetic gynecologic surgery based upon variation of the anatomical appearance of female external genitalia [1]. There are several surgical techniques that have been applied for labia minora labioplasty such as straightforward or partial amputation, central V-plasty (the wedge resection) and its modification, deepithelialized labioreduction, central wedge nymphectomy with a 90-degree Z-plasty, inferior wedge resection and superior pedicle flap reconstruction, and laser labioplasty [2–8]. Existing surgical techniques of clinical applications for labioreduction of the labia minora had been detailed by Ostrzenski elsewhere [9, 10].

Reviewing labium minus labioreduction techniques and performing some of those techniques, Ostrzenski was guided to establish hypothesis that a surgical intervention for labium minus labioreduction should offer reduction of the height and length, should establish symmetry, should preserve natural color and contour of the labium minus, and should restore or create natural appearance of the labium minus frenulum (posterior edge of the fossa navicularis). In the observational prospective, multiple time case series clinical study, this hypothesis was tested, with study's objectives, to develop and to present a newly developed surgical intervention of labium minus labioreduction to determine applicability of this procedure, to evaluate aesthetic surgical outcomes, and to assess potential complications of this procedure. Today, woman's demands for labia minora labioplasty either for aesthetic motives or medical indication(s) are growing; therefore, such a new surgical intervention is very important not only for gynecologist but also for cosmetic-plastic surgeons, urologists, and general surgeons who perform labium minus labioreduction.

## 2. Materials and Methods

Two out of three subjects presented with physical symptoms associated with the labium minus enlargements and one subject presented with dissatisfying appearance of her labia minora. The first subject was a 22-year-old Caucasian woman, G_0_P_0_, unmarried, and a college student, who has been sexually active. The disproportionately protuberant labia minora were responsible for her symptoms such as persistent irritation leading to discomfort during physical activities and following voiding and defecation. Blood flow during menses significantly increased irritations and discomfort due to difficulties in maintaining personal hygiene related to enlargement of the labia minora. Also, she reported superficial dyspareunia, which was caused by twitching and inadvertently pulling the enlarged labia minora into the vaginal pool. Although she learned how to separate her labia minora enough to introduce a penis into her vagina to minimize superficial dyspareunia on insertion, during the act she could not control her discomfort caused by labia minora being brought into the vaginal introitus and the distal vagina.

The second subject was a 27-year-old Caucasian woman, G_3_ P_3003_, married, high school teacher, and sexually active. She requested a surgical reduction of her labia minora for symptomatic enlargement. The symptoms included discomfort associated with rubbing while walking or wearing close-fitting underwear and superficial dyspareunia, which precludes her from reaching an orgasm during sexual intercourse.

The third subject was a 22-year-old Caucasian woman, G_0_P_0_, single, and a professional ballet dancer. She complained about excessively enlarged, significantly protruding, and asymmetrical labia minora. This abnormality forced her to use specially designed compression underwear during her practices and performances. She does not report any physical discomfort when she does not wear compressive underwear on the vulvar area. However, when she wears it, she has significant discomfort, particularly, during her professional dancing. This condition caused a negative body image perception, which led to decreased self-confidence and a social phobia and anxiety. The subject requested to reduce the volume of both labia minora, which she has considered to be responsible for her deteriorated professional, social, and emotional well-beings. Aesthetic dissatisfaction with the subject's external genitalia led to social embracement and emotional disturbances. She felt extreme embarrassment not only during her professional dancing but also during her intimate life due to significant and disproportional overgrown of the labia minora. She presented with the enlarged and asymmetrical in length and height of labia minora. The subject requested to reduce the length and height and to create symmetrical and uniform appearance of the labia minora.

All three women were subjected to newly developed fenestration labioplasty with inferior flap transposition under local infiltration.

A search for the existing literatures was carried out from 1900 to May 2010, using Medical Subject Headings (MeSH) and keywords of fenestration labioplasty, fenestration labioreduction, cosmetic gynecology, labial reduction, labioreduction, labia minora labioplasty, labioplasty, labia minora procedures, female genitalia, labial hypertrophy, vaginal rejuvenation, vaginoplasty, designer vaginoplasty, designer vagina, and labium minus which were selected and used in a search on ISI Web of Science (including conferences proceedings; 1950 PubMed), ACOGNET, ProQuest, OVID, Cochrane Collection, the Lancet on Line Collection, MDConsultant, New England Journal of Medicine, American College of Physician on Line Resources, Highwire Journal, and Citation Index Reference, and a manual search was utilized.

### 2.1. Informed Consent

The Ethics Committee approved the study protocol. An informed consent was structured in accordance with the existing recommendation of the American College of Obstetricians and Gynecologists [11]. A written consent was obtained from each subject of the study. Additionally, all women authorized Ostrzenski to use their clinical data and digital photo images of their genital organs for publishing in medical peer-reviewed journals.

### 2.2. Local Anesthesia

The procedure was executed under local anesthesia without conscious sedation. A thick layer of lidocaine-prilocaine (2.5%/2.5%) cream was applied to the labia minora and immediately adjacent areas bilaterally; the region was covered with sterile gauze for 1 hour, the last 30 minutes before procedures an ice pack was added to this area. Upon removing the ice pack, the remaining anesthetic cream was wiped off and the operative field was prepped with Betadine solution. Half way between the posterior commissure and the upper part of the anus in the middle and the ischopubic ramus, just under the superficial transverse perineal muscle, 5–10 mL of plain 1% lidocaine was injected with the 27 G × 1/2 inch needle and 10 cc syringes (Terumo, Elkton, MD, USA) in one side for local anesthesia. The superficial part of the deep branch of the perineal nerve and the posterior labial nerves were infiltrated with one injection and provided with adequate local anesthesia for this procedures. Neither conscious sedation nor pudendal block was used.

### 2.3. Fenestration Labioreduction with Flap Transposition Technique: Stepwise Approach

**Figure 1:**
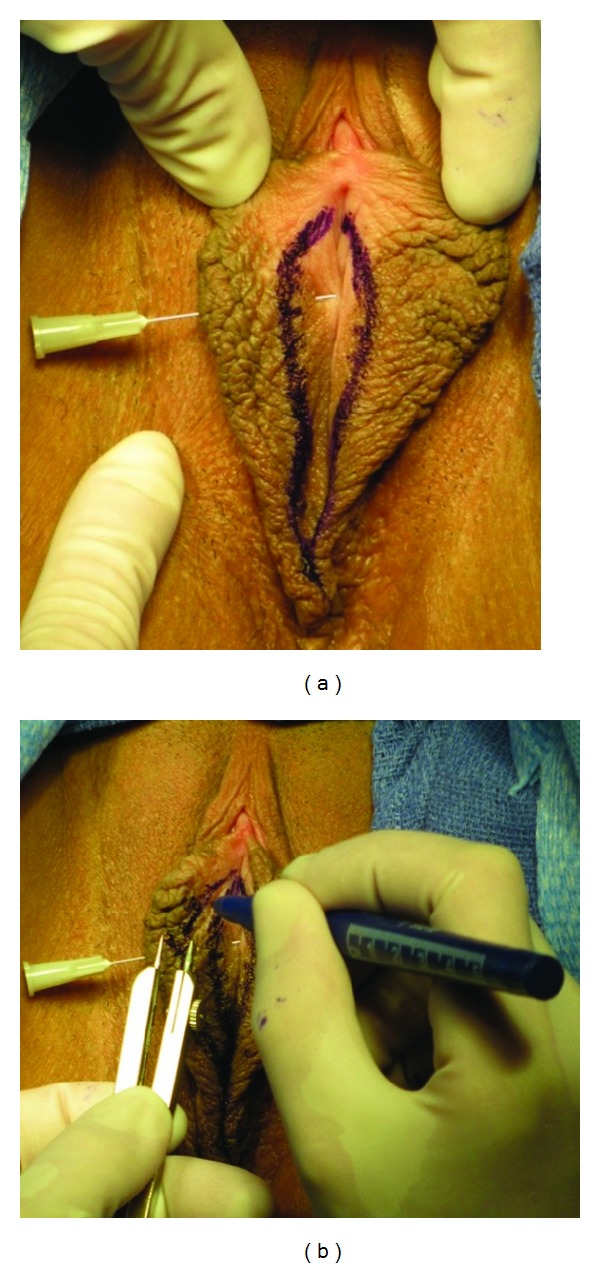
(a) The small needle pierced the labium minus from the inguinal surface (the posterior labium minus) to the vestibular surface (anterior) of the labium. This technique was used to determine the labial base levels, since the posterior base of the labia is higher than anterior labial base. A “helmet shape” marking is shown bilaterally. More tissue needs to be removed from the left labium. (b) The exact measurement of the labia is determined with a caliper.

**Figure 2:**
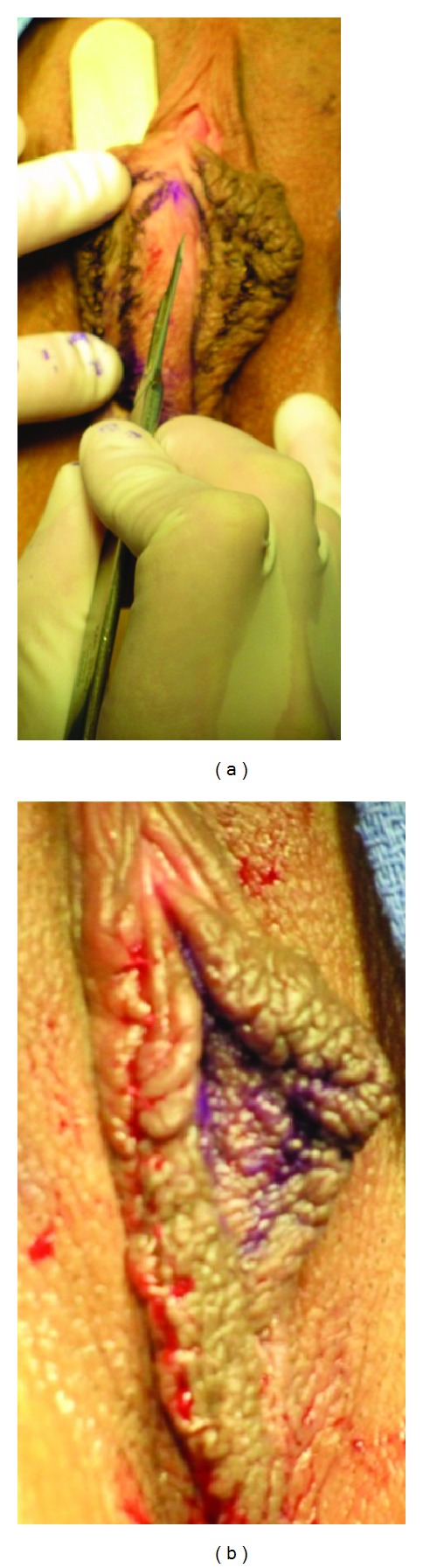
(a) Under the posterior surface of the labia minora, a wooden tongue depressor was placed to protect the labium majus from inadvertent injury and to provide stability for incisions. (b) Right labium minus was reduced in height and length. Suture line is well concealed.

**Figure 3:**
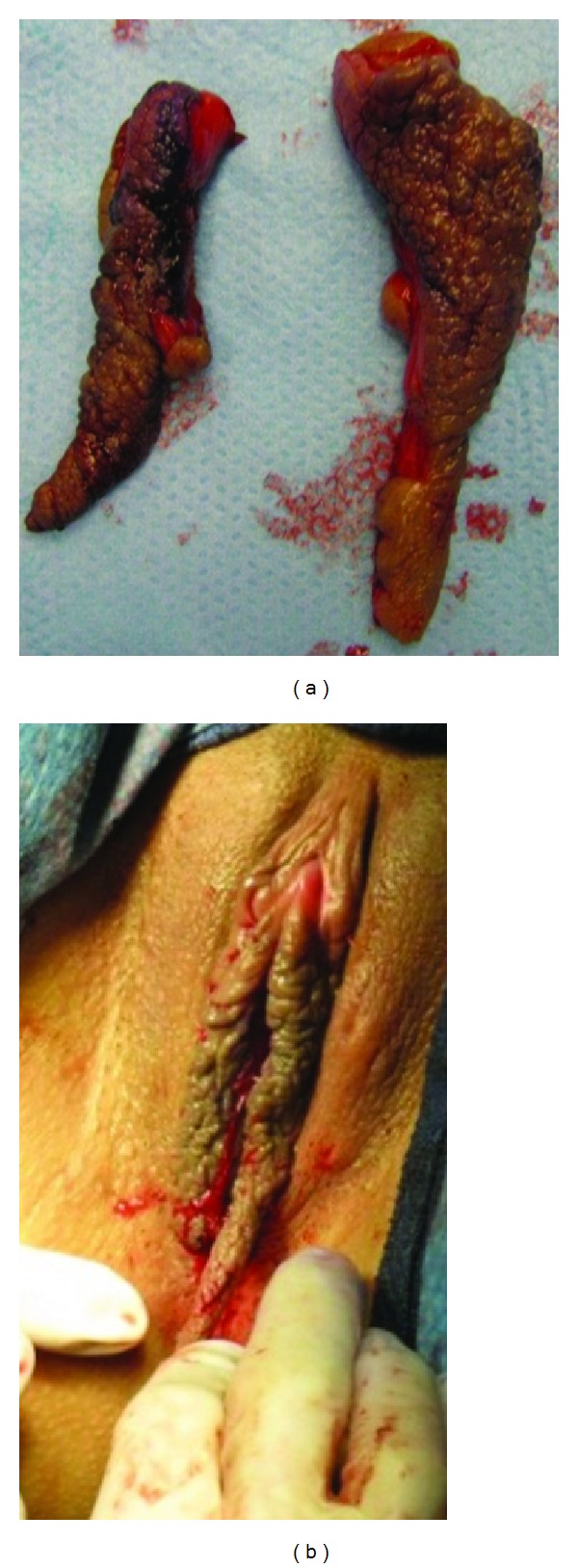
(a) The uneven amount of tissue removed from the left and right labia minora to establish symmetry. (b) The extra length of the inferior flap of labium minus is held in the surgeon's right hand before trimming.

**Figure 4:**
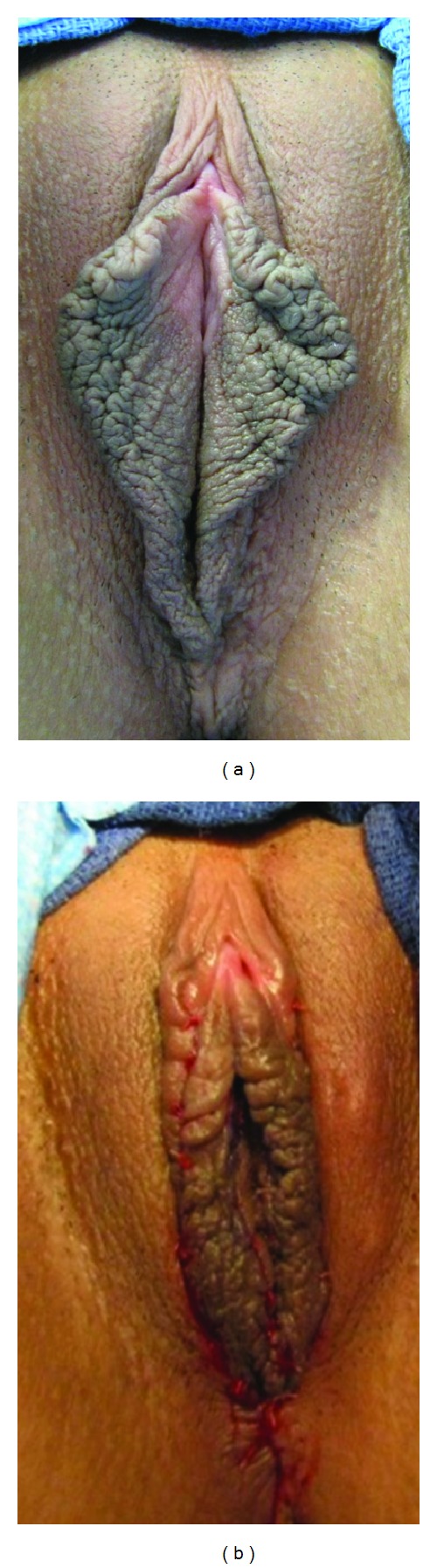
(a) General view of the labia minora before Ostrzenski's fenestration labioplasty with inferior flap transposition. (b) Immediately after the operation. In the right upper part of the labium, the redundant labium minus's fold was resected and reconstructed.

**Figure 5:**
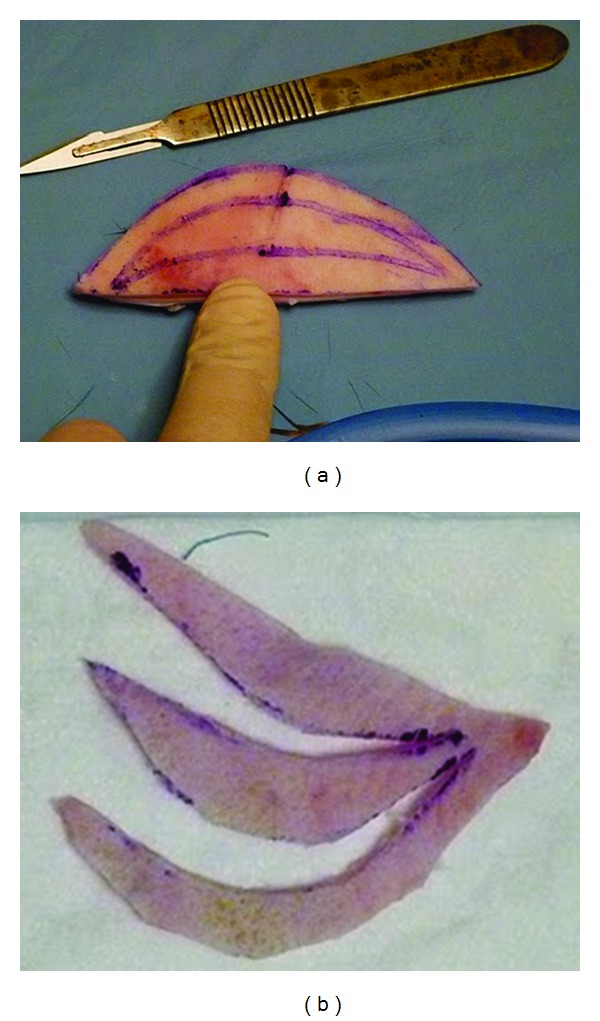
The courtesy of Ostrzenski's “wet” laboratory workshop photos of labium minus fenestration labioreduction (www.cosmetic-gyn.com) used with permission. (a) A bicycle helmet shape marking is depicted on the practicing material. (b) The process of excising the helmet shape.

**Figure 6:**
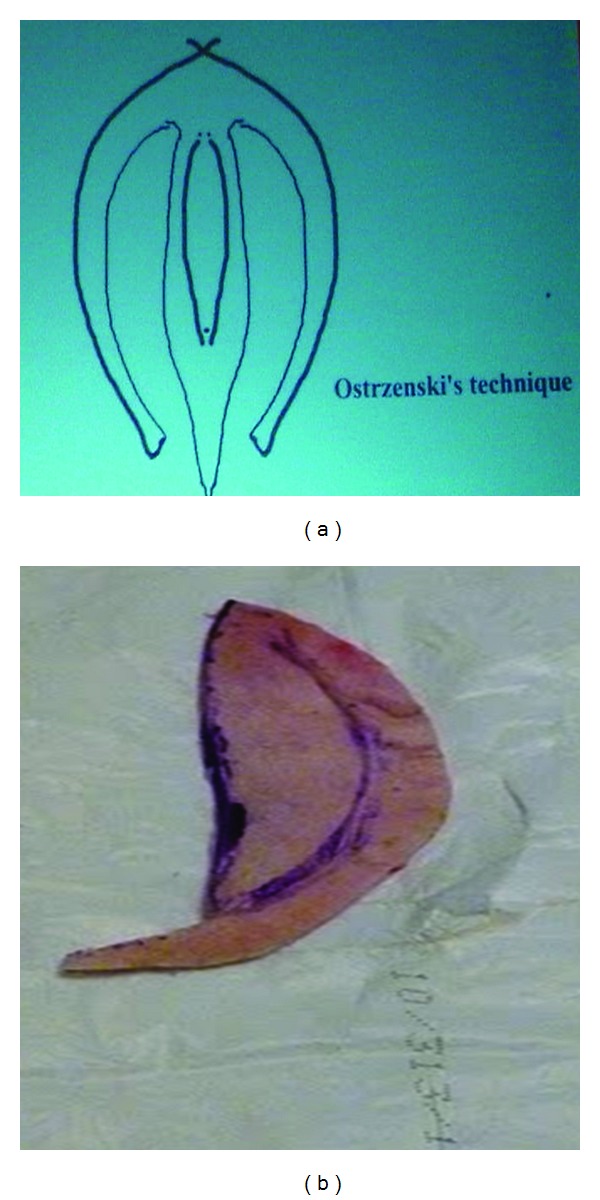
The courtesy of Ostrzenski's “wet” laboratory workshop of labium minus fenestration labioreduction (www.cosmetic-gyn.com) used with permission. (a) Bilaterally, bicycle-shape tissues were resected. The height and the length have been reduced. (b) The inferior flap is ready to be trimmed and connected with the opposite side in the midline.

Upon determining the size of labia minora volume being reduced, the base of the lower margin of incision was determined and outlining of the amount of the tissues being removed was marked in the shape of a “bicycle helmet” within the anterior labial surface; see Figures 1, 5, and 6. Also, in this process the arch of the new labium was determined; see Figure 1. An incision was made with No. 15 surgical blade. The resection in the “bicycle helmet” shape was accomplished and excised, see Figures 2, 5(b), and 6(a). By doing so, the labium was divided into two fragments: the superior strip was partially detached from the rest of the labium and the inferior part at the base of the labium minus. Immediately, the superior strip of the labium was sutured to the lower base edge of the labium minus. Suturing was continued below the newly created arch of the labium. The anterior labial lamina and posterior labial lamina were sutured on both sites separately without suturing the erectile tissues between the labial laminae. The edges were brought together without any tension. The attention was given to the inferior flap transposition. Below the arch, the labium inferior flap is gradually reduced in the wedge shape (the proximal part of the flap being bigger and then the distal segment) gradually getting smaller and thinner to create natural look of the labia. The distal labium part of the inferior flap is modeled in the arch shape to meet in the midline with the opposite distal labium just above the posterior commissure. Such a tissue transposition creates the labium minus frenulum (posterior border of the fossa navicularis). The length of the inferior flap of the labium is trimmed and sutured in the same manner as presented above. The procedure was executed bilaterally in all the subjects; see Figures 1, 2, 3, and 4.

### 2.4. Postoperative Care

Postoperatively, discomfort was controlled with external application of Dermoplast, an antiseptic and pain relieving spray (Medtech, Jackson, WY, USA). Occasionally, subjects as needed took every 4 hours 2 tablets acetaminophen, orally. Sutures were removed on the 7th postoperative day.

## 3. Results

The electronic and manual searches failed to identify fenestration labioreduction with inferior flap transposition or similar surgical intervention. Therefore, this presentation is the first description in the scientific-clinical literature of a fenestration labioreduction with inferior flap transposition technique.

The disproportionately protuberant, enlarged, and asymmetrical labia minora were confirmed in each subject. All subjects reported feelings of decreased body image perception, being sexually inadequate and undesirable, and decreased self-image and confidence.

Two women reported symptoms of persistent irritation leading to discomfort during physical activities, following voiding, defecation, and getting worse during menses as a result of difficulties in maintaining personal hygiene and reported superficial dyspareunia during sexual intercourse. The third subject presented with aesthetic dissatisfaction from her appearance of enlarged, asymmetrical labia minora. The newly developed operation of fenestration labioreduction with inferior flap transposition was applied without intraoperative, short- and long-term complications. In all subjects, the fenestration labioreduction with inferior flap transposition operation reduced the height and length, established symmetry, preserved natural color and contour of the labium minus, and restored or created natural appearance of the labium frenulum (posterior edge of the fossa navicularis). Postoperatively, medical and emotional symptoms and signs resolved; pleasing surgical outcomes exceeded subjects' aesthetic expectations. Additionally, body self-image and confidence improved meaningfully in all subjects. None of the subjects verbally reported feeling of regret and described reduction of the emotional tension, which was generated by conflict of being different and dilemma of feeling of being helpless. Social openness improved and intimate interaction increased, and their body image perception improved following the operation. There were no intraoperative, short-, or long-term complications recorded in all three subjects. Bleeding during the procedure was negligible in all subjects. The average time of surgery measured from the initial incision to completion of fenestration with inferior flap transposition was 36 minutes. Postoperatively, the subjects had uncomplicated recoveries with minimal discomfort. All subjects engaged in vaginal sexual intercourse with their respective male partners 6 weeks following the surgery.

## 4. Discussion

This clinical study's results indicated that practitioners should look at the enlarged labia minora not only for physical symptoms (irritation, difficulties in maintaining personal hygiene, discomfort during physical activities, and discomfort during voiding or defecation) but also from the prospective of sexual dysfunction (pain during vaginal sexual intercourse) or emotional disturbances related to this condition (feeling inadequate, decreased feeling of body image perception, and being embarrassed socially). Therefore, not only clinical symptoms but also an aesthetic aspect plays significant roles in the labium minus enlargement.

Implementation of fenestration labium minus labioreduction with inferior flap transposition demonstrates the use of clinical settings and eliminates potential for denuding the posterior vaginal introitus [12].

Analyzing existing surgical techniques for labioreduction such as central V-plasty, central V-plasty, and central wedge nymphectomy with 90° Z-plasty and W-plasty one can draw a conclusion that these surgical techniques will leave transverse single or multiple scars on longitudinal organ such as the labium minus [2, 4, 7]. Therefore, transverse scaring effectively minimizes implementation of these procedures. An inferior wedge resection technique leaves completely denuded areas around posterior and lateral vaginal introitus, which lead to high superficial dyspareunia and unaccepted high rates of wound separations [5, 8]. Consequently, this technique will not only compromise aesthetic outcomes but also can be responsible for sexual dysfunction (superficial dyspareunia).

Other labioreduction surgical interventions such as labial partial amputation, deepithelialized reduction labioplasty, inferior wedge resection and superior pedicle flap reconstruction will be appropriate for clinical implementations [2, 3, 6, 8]. Liao et al. and Ostrzenski presented detailed evaluation of each surgical technique relating to labioreduction of the labia minora [9, 10, 13].

Differences between existing surgical procedures and the fenestration labioreduction with inferior flap transposition (FLFT) technique are significant. None of existing surgical interventions will encompass reduction of the height and length, established symmetry, preserved natural color and contour of the labia minora, and restored or created natural appearance of the labium frenulum (posterior edge of the fossa navicularis) in one procedure and only the fenestration labioreduction with inferior flap transposition technique incorporates all of them. When compared to partial labial amputation, FLFT preserved natural color and contour of the labium minus and restored or created natural appearance of the posterior edge of the fossa navicularis and partial amputation does not [2, 3]. Deepithelialized reduction labioplasty can only be offered for very thin and elongated labia minora, since it makes the labia minora much thicker or bulky at the base and FLFT will not do it [6]. Deepithelialized reduction labioplasty can only reduce the height of the labia minora; FLFT will reduce both the height and the length of the labia minora [6]. Deepithelialized reduction labioplasty will not restore or create the posterior boarder of the fossa navicularis and FLFT will [6].

The inferior wedge resection and superior pedicle flap reconstruction will not restore or create the posterior boarder of the fossa navicularis and FLFT will [8]. The inferior wedge resection and superior pedicle flap reconstruction will create unnatural appearance of the proximal labia connection and FLFT will not [8]. The inferior wedge resection and superior pedicle flap reconstruction has tendency to stretch the superior pedicle flap and FLFT has not [8]. The inferior wedge resection and superior pedicle flap reconstruction often creates undesirable permanent wrinkling and irregularity at the proximal approximation of the incision and the FLFT procedures are free of it [8].

By all means, Ostrzenski does not make any suggestion that FLFT is the only procedure that should be used in all cases. Contrary, there is no study to support which procedure is superior over others. Therefore, the clinical judgment should be exercise, and all four relevant procedures (labial partial amputation, deepithelialized reduction labioplasty, and inferior wedge resection and superior pedicle flap reconstruction, and fenestration labioreduction with inferior flap transposition) should be taken into account and the surgical intervention which suits patient's needs should be selected.

Small power of the study can be considered as a weakness; however, to test the established hypothesis the numbers of cases were sufficient to determine surgical applicability of FLFT. The importance of this study's results strongly suggest that not only aesthetic pleasing results can be accomplished in well-selected women by applying this surgical intervention but also the results imply that the clinical symptoms as well as emotional disturbances related to the enlarged labium minus can be eradicated.

## 5. Conclusions

In this study group, fenestration labioreduction with inferior flap transposition surgical intervention can be executed effortlessly without complications and the method can be reproduced; aesthetically, the new operation achieves very pleasing results and the procedure improves physical-, emotional, and social well being.

## References

[1] ACOG Committee Opinion Number 378 ‘Vaginal rejuvenation’ and cosmetic vaginal procedures.

[2] Radman HM (1976). Hypertrophy of the labia minora. *Obstetrics and Gynecology*.

[3] Hodgkinson DJ, Hait G (1984). Aesthetic vaginal labioplasty. *Plastic and Reconstructive Surgery*.

[4] Alter GJ (1998). A new technique for aesthetic labia minora reduction. *Annals of Plastic Surgery*.

[5] Rouzier R, Louis-Sylvestre C, Paniel B-J, Haddad B (2000). Hypertrophy of labia minora: experience with 163 reductions. *American Journal of Obstetrics and Gynecology*.

[6] Choi HY, Kim KT (2000). A new method for aesthetic reduction of labia minora (the deepithelialized reduction labioplasty). *Plastic and Reconstructive Surgery*.

[7] Giraldo F, González C, de Haro F (2004). Central wedge nymphectomy with a 90-degree Z-plasty for aesthetic reduction of the labia minora. *Plastic and Reconstructive Surgery*.

[8] Munhoz AM, Filassi JR, Ricci MD (2006). Aesthetic labia minora reduction with inferior wedge resection and superior pedicle flap reconstruction. *Plastic and Reconstructive Surgery*.

[9] Ostrzenski A (2011). Cosmetic gynecology in the view of evidence-based medicine and ACOG recommendations: a review. *Archives of Gynecology and Obstetrics*.

[10] Ostrzenski A (2013). Selecting aesthetic gynecologic procedures for plastic surgeons: a review of target methodology. *Aesthetic Plastic Surgery*.

[11] The American College of Obstetricians and Gynecologists (2008). *Compendium of Selected Publications*.

[12] Ostrzenski A (2011). The first clinical classification of vaginal introital defects. *European Journal of Obstetrics Gynecology and Reproductive Biology*.

[13] Liao L-M, Michala L, Creighton SM (2010). Labial surgery for well women: a review of the literature. *An International Journal of Obstetrics and Gynaecology*.

